# Amoebic Liver Abscess: Rare Entity in Recent Times

**DOI:** 10.7759/cureus.17698

**Published:** 2021-09-03

**Authors:** Ravikanth Reddy

**Affiliations:** 1 Radiology, St. John's Hospital, Bengaluru, IND

**Keywords:** endemic regions, serology, ultrasonography, amoebic liver abscess, entamoeba histolytica

## Abstract

Intestinal amoebiasis and sequelae such as amoebic liver abscess are rarely reported in the era of modern medicine. Atypical presentation of amoebic liver abscess with high false-positive results on serology in endemic regions poses major diagnostic issues in the developing nations of the world. We report a case of amoebic liver abscess and describe the imaging appearances in a 59-year-old female. A detailed medical history was elicited to determine the etiology of amoebic liver abscess. Furthermore, the diagnosis was confirmed based on serological tests. Percutaneous aspiration of the amoebic liver abscess was performed, and treatment was initiated with intravenous metronidazole followed by diloxanide furoate. The patient has been on follow-up since three months with a negative stool examination and with no complaints of recurrence.

## Introduction

Amoebiasis is a parasitic infection caused by *Entamoeba histolytica*. Precarious lifestyle and low socioeconomic status have been advocated as some of the predisposing factors for the development of amoebic liver abscess. The infection begins with ingestion of water or food contaminated with cysts of *E. histolytica* which presents as colitis leading to systemic spread with sequelae such as amoebic liver abscess, hepatopulmonary fistula, amoebic pericarditis and cerebral abscess [1]. Ultrasonography with a sensitivity of more than 90% and high diagnostic accuracy has been highly recommended as the initial imaging investigation of choice for the diagnosis of amoebic liver abscess [2]. Ultrasonography is an essential imaging modality for prompt and accurate diagnosis of the entity and for monitoring lesion progression and timely detection of potential complications. Literature review suggests that therapeutic drainage of amoebic liver abscess is required in 80%-90% of cases [3]. We have provided the diagnostic approach for amoebic liver abscess in resource starved settings and endemic regions of developing nations.

## Case presentation

**Figure 1:**
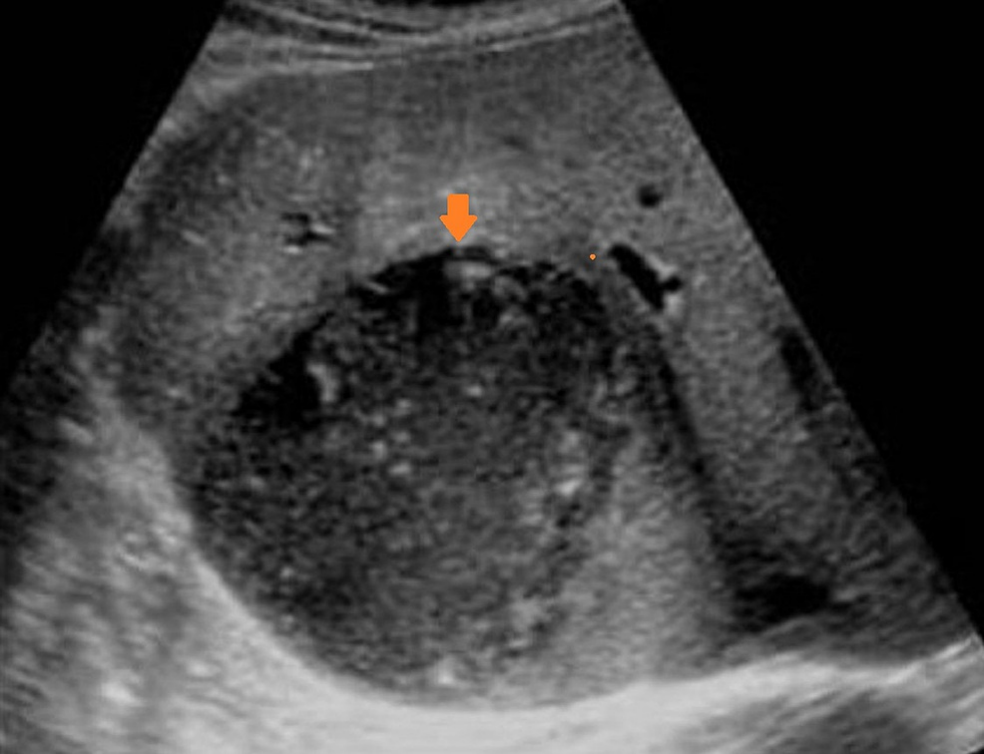
Longitudinal ultrasonography image demonstrating a well-defined collection (arrow) with a hypoechoic wall and internal contents that shows fine low-level echoes consistent with features of amoebic liver abscess. Note the distal acoustic enhancement posterior to the lesion in the right lobe of liver.

**Figure 2:**
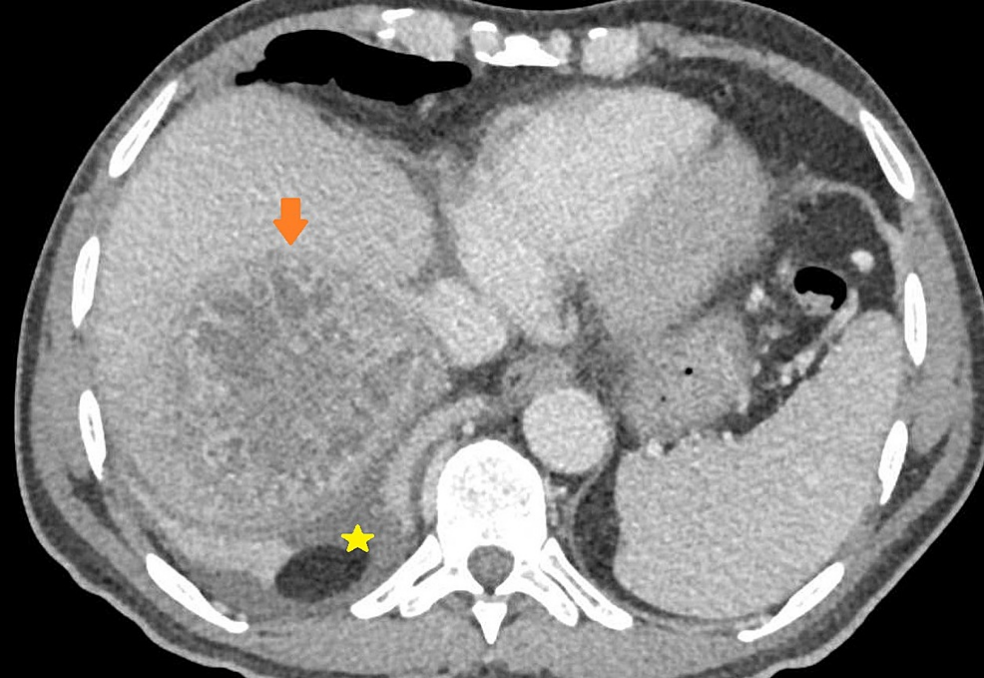
Contrast-enhanced computed tomography image of the upper abdomen in portal venous phase demonstrating a well-circumscribed collection (arrow) in segments 5 and 6 of the right lobe of liver with ragged edges and multiple internal septations. Note the localized perihepatic fluid collection (star) due to subcapsular extension of the abscess.

**Figure 3:**
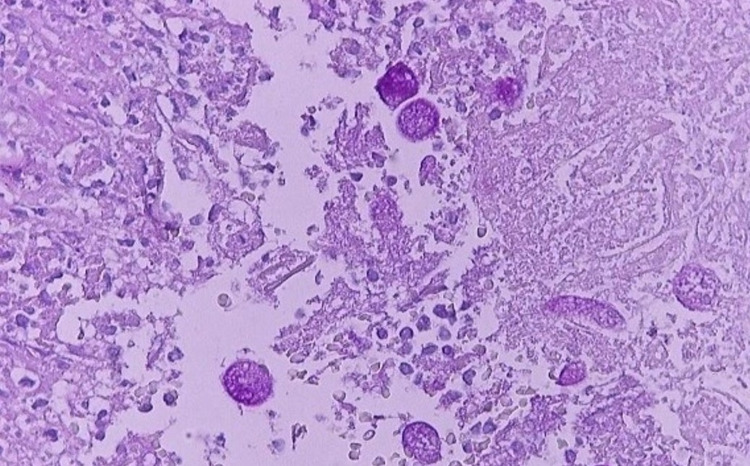
Histopathology image demonstrating positivity to Periodic Acid Schiff staining. Note the hepatocytes with dense inflammatory infiltrate interspersed with areas of necrosis and several amoebic trophozoites (Periodic Acid Schiff stain, 400x magnification).

A 59-year-old woman presented to emergency department with complaints of insidious onset pain in the right hypochondrium, vomiting and nonresolving fever for two weeks. Two months prior, the patient made a trip to Bangladesh for a period of six weeks. Clinical examination revealed mild hepatomegaly with tenderness in the right hypochondrium. There was no history of jaundice. The patient had tachycardia and tachypnea with normal oxygen saturation levels. Blood investigations revealed elevated C-reactive protein at 127 (normal range: <5 mg/l) and white blood cell count at 18.3 (normal range: 4-11×109/l). Liver function test results were deranged with a total bilirubin of 13 (normal range: 0-15 mmol/l), albumin of 24 (normal range: 35-50 g/l), alanine aminotransferase of 67 (normal range: 5-37 U/l), alkaline phosphatase of 142 (normal range: 40-120 U/l) and γ-glutamyl transpeptidase of 62 (normal range: 0-50 u/l). Transabdominal ultrasonography revealed mild hepatomegaly with evidence of a well-defined predominantly hypoechoic lesion measuring 6 x 6 cm in segments 5 and 6 of the right lobe of liver with evidence of a hypoechoic wall and internal contents that shows fine low-level hypoechoic echoes but no vascularity on color Doppler (Figure 1). Based on ultrasonography findings, differentials included amoebic liver abscess, pyogenic abscess, hepatocellular carcinoma with central necrotic core and hepatic metastases. Contrast-enhanced computed tomography of the upper abdomen demonstrated a well-circumscribed nonenhancing abscess measuring 6 cm in diameter and involving segments 5 and 6 of the right lobe of liver. Furthermore, there was subcapsular extension with marginal liver interface adjacent to the region of right hemidiaphragm (Figure 2). Pigtail catheter drainage was attempted, and the drained fluid was sent for microscopy and culture sensitivity (Figure 3). Positive serological testing for amoebiasis confirmed the imaging diagnosis. Furthermore, indirect fluorescent antibody (IFA) test returned an initial titer of 1:128 which eventually increased four-fold in five days. A diagnosis of amoebic liver abscess was confirmed, and treatment was initiated with intravenous metronidazole supplemented with diloxanide furoate, following which the patient showed dramatic clinical improvement. Follow-up period for three months was uneventful with stool examination negative for cysts at the end of three months.

## Discussion

Amoebic liver abscess develops secondary to infection by the protozoan *E. histolytica*. Atypical presentation of amoebic liver abscess with high false-positive results on serology in endemic regions poses major diagnostic issues in the developing nations of the world [4]. On ultrasonography, the characteristics of amoebic liver abscess are rounded configuration, homogenous low-level internal echoes, lack of wall echoes, location contiguous with the liver capsule and distal acoustic enhancement [5]. Additional findings of amoebic liver abscess on ultrasonography include incomplete rim of edema, the margins of the abscess tend to be smooth in 60% of cases and nodular in 40% of cases [6], internal septations present in 30% of cases [7], and focal intrahepatic biliary radicle dilatation peripheral to the abscess noted in minority of cases. Extrahepatic findings include perihepatic fluid collection, right-sided pleural effusion, gastric or colonic involvement and retroperitoneal extension [8]. Differential diagnosis for amoebic liver abscess is hydatid cyst, pyogenic abscess and solid mass lesion with necrosis [9]. However, differentiation of amoebic liver abscess from a pyogenic abscess is difficult based only on clinical investigations and imaging techniques. Serological tests are highly specific and sensitive for amoebic liver abscess [10]. Multiplicity is less commonly encountered in amoebic liver abscess than in pyogenic abscess [11]. Concomitant intestinal amoebiasis may hold the clue for amoebic liver abscess [12]. Hepatocellular carcinoma with spontaneous necrosis appears heteroechoic with a peripheral hypoechoic halo and demonstrates hypervascularity on ultrasonography [13]. Most importantly, clinicians should also be aware of the epidemiology of the disease, as to correlate patients presenting symptoms with the clinical diagnosis, which is very much applicable to endemic cases of amoebiasis.

## Conclusions

This case report describes the ultrasonography appearances of amoebic liver abscess which is a rare entity encountered in elderly patients that warrants prompt and urgent treatment, and the entity should be distinguishable from other causes of abscess formation in the liver. In conclusion, ultrasonography can be used as an initial imaging investigation of choice for preprocedural assessment of patients with amoebic liver abscess, considering the treatable nature of the entity and potentially devastating outcome of an untreated abscess. Utilization of high-resolution ultrasonography supplemented by serological and molecular testing may eliminate the diagnostic dilemma in atypical presentations of amoebic liver abscess. Radiologists and sonologists especially from endemic regions must be aware of the ultrasonography features of amoebic liver abscess to confidently diagnose the entity in suspected cases of fever with a travel history especially to countries of the Middle East and Eastern Asia.
